# Prediction of fluid responsiveness in mechanically ventilated cardiac surgical patients: the performance of seven different functional hemodynamic parameters

**DOI:** 10.1186/s12871-018-0520-x

**Published:** 2018-05-22

**Authors:** Michael T. Ganter, Martin Geisen, Sonja Hartnack, Omer Dzemali, Christoph K. Hofer

**Affiliations:** 1 Institute of Anesthesiology, Kantonsspital Winterthur, Brauerstr. 15, 8401 Winterthur, Switzerland; 2 Institute of Anesthesiology and Intensive Care Medicine, Triemli City Hospital Zurich, Birmensdorferstr. 497, 8063 Zurich, Switzerland; 3 Section of Epidemiology, Vetsuisse Faculty, University of Zurich, Winterthurerstr. 270, 8057 Zurich, Switzerland; 4 Division of Cardiac Surgery, Triemli City Hospital Zurich, Birmensdorferstr. 497, 8063 Zurich, Switzerland

**Keywords:** Stroke volume variation, Pulse pressure variation, Pleth variability index, Functional hemodynamic parameter, Pulse wave analysis

## Abstract

**Background:**

Functional hemodynamic parameters such as stroke volume and pulse pressure variation (SVV and PPV) have been shown to be reliable predictors of fluid responsiveness in mechanically ventilated patients. Today, different minimally- and non-invasive hemodynamic monitoring systems measure functional hemodynamic parameters. Although some of these parameters are described by the same name, they differ in their measurement technique and thus may provide different results. We aimed to test the performance of seven functional hemodynamic parameters simultaneously in the same clinical setting.

**Methods:**

Hemodynamic measurements were done in 30 cardiac surgery patients that were mechanically ventilated. Before and after a standardized intravenous fluid bolus, hemodynamics were measured by the following monitoring systems: PiCCOplus (SVV_PiCCO_, PPV_PiCCO_), LiDCO_rapid_ (SVV_LiDCO_, PPV_LiDCO_), FloTrac (SVV_FloTrac_), Philips Intellivue (PPV_Philips_) and Masimo pulse oximeter (pleth variability index, PVI). Prediction of fluid responsiveness was tested by calculation of receiver operating characteristic (ROC) curves including a gray zone approach and compared using Fisher’s Z-Test.

**Results:**

Fluid administration resulted in an increase in cardiac output, while all functional hemodynamic parameters decreased. A wide range of areas under the ROC-curve (AUC’s) was observed: AUC-SVV_PiCCO_ = 0.91, AUC-PPV_PiCCO_ = 0.88, AUC-SVV_LiDCO_ = 0.78, AUC-PPV_LiDCO_ = 0.89, AUC-SVV_FloTrac_ = 0.87, AUC-PPV_Philips_ = 0.92 and AUC-PVI = 0.68. Optimal threshold values for prediction of fluid responsiveness ranged between 9.5 and 17.5%. Lowest threshold values were observed for SVV_LiDCO_, highest for PVI.

**Conclusion:**

All functional hemodynamic parameters tested except for PVI showed that their use allows a reliable identification of potential fluid responders. PVI however, may not be suitable after cardiac surgery to predict fluid responsiveness.

**Trial registration:**

NCT02571465, registered on October 7th, 2015 (retrospectively registered).

## Background

The assessment of the patient’s fluid status is a key element in perioperative care. The overall goal is to keep patients in a steady fluid state and avoid any disturbance like hyper- or hypovolemia. Inadequate fluid therapy is associated with impaired patient’s outcome. Therefore, fluids should only be given in well-defined protocols according to individual needs. Like any other medication, fluid therapy must neither be overdosed nor underdosed [1].

Before any fluid administration, patients have to be clinically assessed. Obvious fluid deficits have to be immediately corrected in acute circulatory failure without any prior evaluation of preload responsiveness. In other clinical settings however, fluids should only be given to patients with a predicted positive fluid response – this means that patients should be tested before fluid administration [2]. Thereby, a change in preload is provoked while monitoring subsequent changes in stroke volume (SV) or its surrogates such as pulse pressure (PP). When the patient operates on the steep portion of the Frank-Starling curve, SV or its surrogates will change significantly. Thus, the prediction of fluid responsiveness is positive and a relevant increase in SV will happen upon fluid loading [3].

Over the last years, functional hemodynamic parameters have become well established to predict fluid responsiveness in mechanically ventilated patients. Positive pressure mechanical ventilation induces a cyclic reduction in left ventricular preload mainly through a decrease in venous return. The change in preload throughout the respiration cycle becomes significant in hypovolemia. Thereby, these cyclic changes in preload during respiration may result in variations of SV (SVV) and PP (PPV) [4].

A major issue with fluid responsiveness is what exact values to measure and how, and many different technologies have been developed and tested over the last years in an attempt to find the ideal technique [5]. Even less invasive methods like pulse oximeters increasingly report such functional hemodynamic parameters. Only few data exist on cross-comparison of these technologies. Therefore, the aim of our study was to evaluate these different technologies in the same clinical setting and to compare their performance in predicting fluid responsiveness.

## Methods

### Patients

With local ethics committee approval, adult patients scheduled for elective coronary artery bypass grafting (CABG) were included in this study after obtaining their written informed consent. The study was performed postoperatively in the intensive care unit (ICU). Patients undergoing emergent cardiac surgery, aged younger than 18 years, and those with with impaired left-ventricular function (EF < 45%), arrhythmias, intraventricular shunts, intra-aortic balloon counterpulsation or severe peripheral arterial occlusive disease were excluded.

### Monitoring

In all patients a standard monitoring (IntelliVue MP70, Philips Medical Systems, Philips Healthcare, 5680 DA Best, Netherlands) was used that included 5-lead ECG, pulse oximetry, and central venous pressure (CVP) via a central venous line inserted into the right internal jugular vein. Invasive arterial blood pressure was assessed via femoral artery access by a 5-French thermistor tipped catheter (Pulsiocath, Pulsion Medical Systems, Munich, Germany), which was connected to a PiCCO_2_ monitor. The monitor was calibrated using transpulmonary thermodilution according to manufacturer’s recommendations using triplicate injections of 15 ml ice-cold saline. An additional cannula was placed into the left radial artery that was connected with a FloTrac sensor attached to the Vigileo system (Software Version 3.02; Edwards Lifesciences, Irvine, CA USA) and an analogue pressure output module of the IntelliVue System was pluged with the LiDCO_Rapid_ monitoring system (LiDCO Ltd., London, UK). All systems were zeroed at mid-axillary. Signal quality was tested by visual arterial waveform assessment and square-wave flush test. A Masimo pulse oximeter (Masimo Coorporation, Irvine, Ca, USA) was attached with its finger cuff to the third or fourth finger on the hand opposite the side of the radial artery catheter. Detailed aspects of all monitoring systems releated to technical issues and especially algorithms used for assessment of stroke volume and functional hemodynamic parameters have been reported in detail elsewhere [6–8]*.*

### Patient management

Postoperative management was performed according to institutional standards. The patients remained sedated during the study period. Propofol (1–2 mg kg^− 1^ h^− 1^), remifentanil (2–5 μg kg^− 1^ h^− 1^) and rocuronium (0.2–0.5 mg kg^− 1^ h^− 1^) was given to allow complete mechanical ventilation. A volume-controlled mode was applied with a tidal volume of 8–10 ml kg^− 1^ and a respiratory rate of 12 min^− 1^ aimed at normoventilation (pCO_2_ at 4.0–4.5 kPa).

### Study protocol

After arrival in the ICU all monitoring devices were installed. Study measurements were started when the physician in charge made the decision to give i.v. fluids. The decision to give i.v. fluid was based on the presence of at least one clinical sign of acute circulatory failure or associated signs of hypoperfusion. Before fluid administration, hemodynamic data of the IntelliVue monitor – heart rate (HR), mean arterial pressure (MAP), pulse pressure variation (PPV_Philips_) and CVP were recoreded as well as data of the PiCCO_2_ system, i.e. cardiac output (CO), stroke volume (SV), stroke volume variation (SVV_PiCCO_) and PPV_PiCCO_. Moreover, data of the FloTrac/Vigileo (SVV_FloTrac_), the LiDCO_Rapid_ monitor (SVV_LiDCO_, PPV_LiDCO_) and the Masimo device (pleth variability index, PVI) were measured. All hemodynamic measurements were repeated after the administration of a standardized i.v. fluid bolus (500 mL of gelatine solution given over a 20 min time period; Physiogel® balanced, B. Braun AG, Melsungen, Germany).

### Statistical analysis

Statistical analysis was done using Microsoft Excel (Version 12.3.2 for MAC 2008, Microsoft Corporation, Redmond, WA), Sigmaplot (Version 12.0, Systat, San Jose CA), SPSS® 10.0 (SPSS® Inc., Chicago, IL) and software R [9], a language and environment for statistical computing and graphics (with the packages “pROC” and “Diagnosis Med” [10]). Based on the assumption of α = 0.05, power = 0.8, a normal distribution and a standard deviation of stroke volume = 20% as well as clinical relevant different changes of stroke volume = 15% induced by fluid loading, a sample size of 30 was calculated (including two patients as potential drop-outs). Paired t-test was used for comparison of hemodynamic data before and after fluid administration. Prediction of fluid responsiveness was assessed by calculating the area under the receiver operating characteristic (ROC) curve for a stroke volume increase > 15% and corresponding threshold values were determined. Gray zones were obtained using the following approaches [11, 12]: Based on 2000 bootstrap samples 95% confidence intervals of the best threshold (Youden index) were determined. Non-parametric and binormal ROC curves were estimated including a diagnosis tolerance of 10%. Thus, test results with a sensitivity and specificity below 90% are considered to be in the inconclusive zone, i.e. the gray zone. The widest gray zone was obtained as the final gray zone according to previously established and published methods [11, 12]. ROC curves were compared by Fisher’s Z-test. A *P* - value < 0.05 was considered to be statistically significant. Unless otherwise stated, data are presented as mean ± standard deviation (SD).

## Results

### Study population

The study was performed in 30 patients in the postoperative period after elective CABG: 4 ± 1 by-passes were done, EuroSCORE (European System for Cardiac Operative Risk Evaluation, [13]) was 3.5 ± 2.0, 6 patients were female (20%), mean age was 65.5 ± 8.3 years, body mass index was 27.2 ± 4.8 kg m^− 2^, all patients had a preserved left ventricular function, and postoperative ejection fraction assessed by transesophageal echocardiography was 64.2 ± 6.5%.

### Hemodynamic measurements

Intravenous fluid administration resulted in significant hemodynamic changes: There was an increase of MAP, CVP and CO, whereas all functional hemodynamic parameters (PPV_Philips_, SVV_PiCCO_, PPV_PiCCO_, SVV_FloTrac_, SVV_LiDCO_, PPV_LiDCO_ and PVI) decreased (Table 1). Mean SV increase was 19.2 ± 12.7%; a stroke volume increase of > 15% was observed in 17 patients (57%).

**Table 1:** Hemodynamic parameters

		Before fluid administration	After fluid administration	*p*
HR	*beats min* ^*− 1*^	86 ± 7	86 ± 8	0.550
MAP	*mmHg*	78.3 ± 10.4	85.1 ± 10.4	0.012
CVP	*mmHg*	9.1 ± 3.2	11.0 ± 3.9	< 0.001
CO	*Lmin*	5.0 ± 1.0	6.1 ± 1.1	< 0.001
SV	*ml*	59.0 ± 14.8	73.0 ± 17.1	< 0.001
PPV_Philips_	*%*	17.2 ± 7.1	9.5 ± 4.4	< 0.001
SVV_PiCCO_	*%*	18.7 ± 7.4	11.9 ± 5.4	< 0.001
PPV_PiCCO_	*%*	17.3 ± 7.1	10.7 ± 4.9	< 0.001
SVV_FloTrac_	*%*	16.4 ± 5.3	10.0 ± 3.8	< 0.001
SVV_LiDCO_	*%*	15.5 ± 6.7	9.2 ± 4.0	< 0.001
PPV_LiDCO_	*%*	18.5 ± 7.7	11.2 ± 4.8	< 0.001
PVI	*%*	19.0 ± 8.6	12.8 ± 5.4	< 0.001

*CO* Cardiac output, *HR* Heart rate, *MAP* Mean arterial pressure, *PPV* Pulse pressure variation, *PVI* Pleth variability index, *SV* Stroke volume, *SVV* Stroke volume variation

### Prediction of fluid responsiveness and comparison of functional parameters

**Fig. 1:**
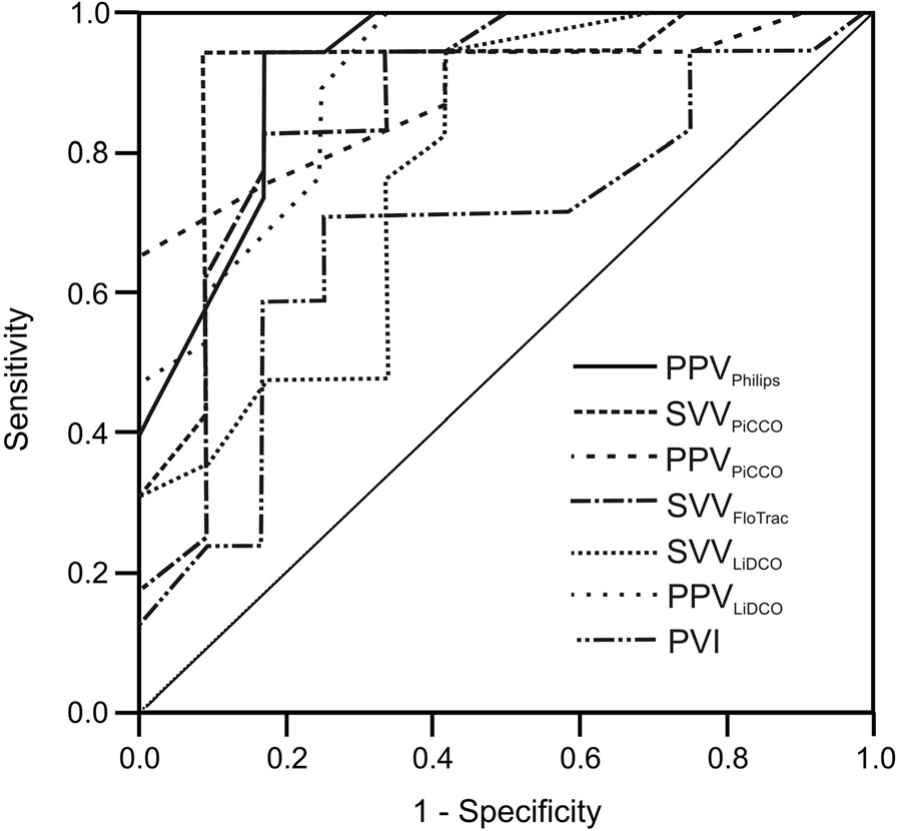
Prediction of fluid responsiveness by receiver-operating characteric (ROC) curves. PPV = Pulse pressure variation, PVI = Pleth variability index, SVV = Stroke volume variation

**Fig. 2:**
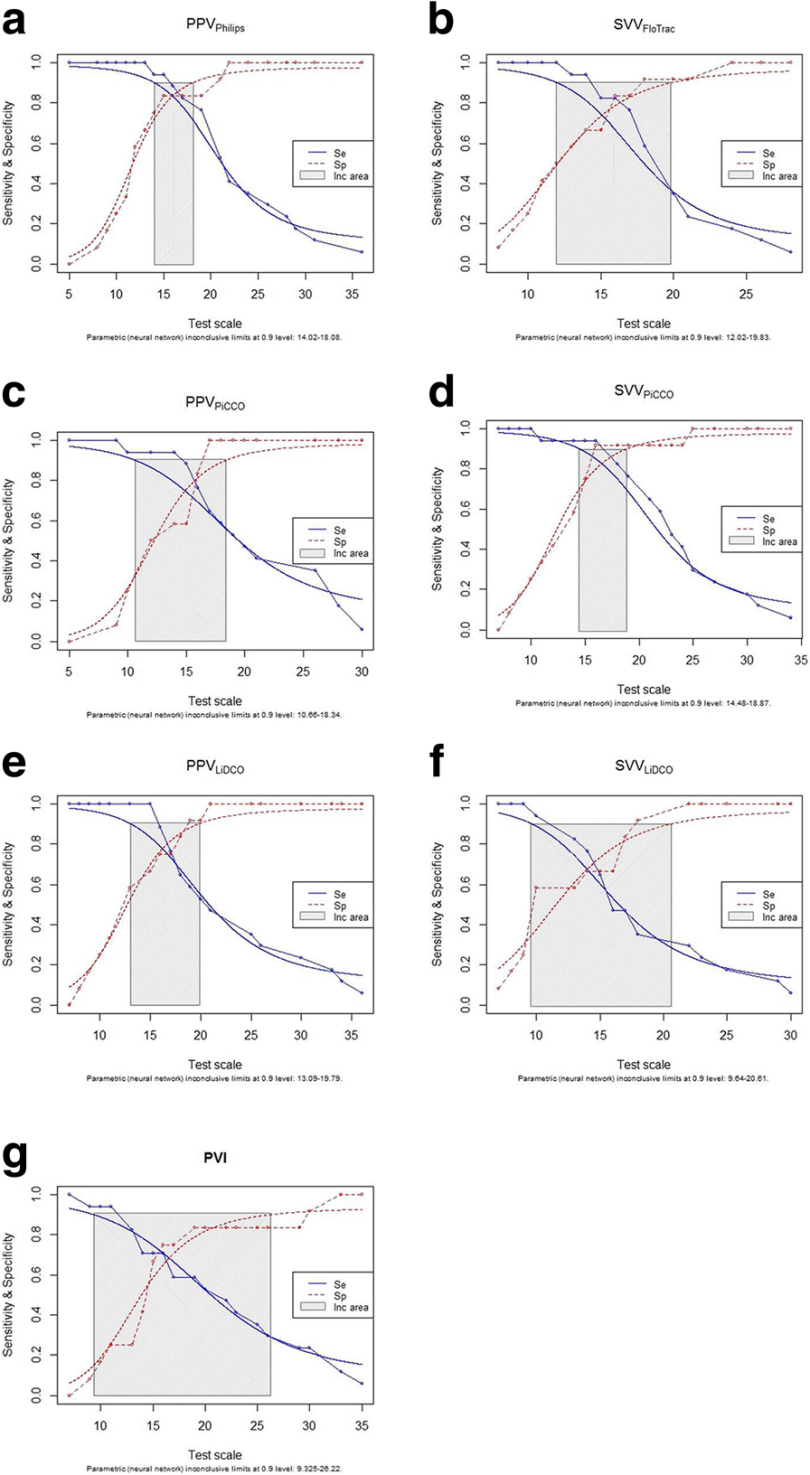
Gray zone determination for the seven functional hemodynamic parameters.Two-graph ROC curves: sensitivity (Se), specificity (Sp), inconclusive zone (Inc area) = gray zone, represented as hatched rectangles. Test results with a sensitivity and specificity below 90% are considered to be in the inconclusive zone. Technologies used: Philips Intellivue (A), FloTrac (B), PiCCOplus (C, D), LiDCO_rapid_ (E, F), and Masimo pulse oximeter (G). PPV = Pulse pressure variation, PVI = Pleth variability index, SVV = Stroke volume variation

ROC curve analysis revealed areas under the curves (AUC) between 0.87 and 0.92 for PPV_Philips_, SVV_PiCCO_, PPV_PiCCO_, SVV_FloTrac_ and PPV_LiDCO_, while AUC for SVV_LiDCO_ and PVI was 0.78 and 0.68, respectively (Fig. 1, Table 2). Optimal threshold values ranged between 9.5 and 17.5%. Lowest threshold was observed for SVV_LiDCO_, highest for PPV_PiCCO_, PPV_LiDCO_ and PVI (Table 2). Gray zones were calculated with the two different statistical approaches (stratified boodstrap replicates, 10% diagnosis tolerance) and the widest gray zone was obtained as the final gray zone according to previously established and published methods [11, 12]. Smallest gray zone was obtained for SVV_PiCCO_ (13.5–18.9%) and PPV_Philips_ (14.1–18.0%), the largest gray zones were found for SVV_LiDCO_ (9.6–20.6%) and PVI (9.3–26.2%) (Fig. 2, Table 2).

**Table 2:** Prediction of fluid responsiveness

	AUC	95% CI	*p*	Threshold A	95% CI	Threshold B	95% CI
PPV_Philips_	0.92	0.81/1.00	< 0.001	14.5	12.0/16.5	15.9	14.1/18.0
SVV_PiCCO_	0.91	0.79/1.00	< 0.001	15.5	15.0/17.0	16.5	14.5/18.9
PPV_PiCCO_	0.88	0.61/0.95	0.001	16.5	12.0/17.5	16.0	10.7/18.3
SVV_FloTrac_	0.87	0.73/0.97	0.001	15.5	11.5/17.5	14.6	12.0/19.8
SVV_LiDCO_	0.78	0.61/0.96	0.012	9.5	9.5/20.0	13.5	9.6/20.6
PPV_LiDCO_	0.89	0.78/0.99	< 0.001	14.0	13.0/20.5	16.4	13.2/19.7
PVI	0.68	0.48/0.89	0.097	15.5	10.5/19.5	17.5	9.3/26.2

*AUC* Area under the curve, *CI* Confidence interval, *PPV* Pulse pressure variation, *PVI* Pleth variability index, *SVV* Stroke volume variation, *Threshold A* Gray zone method: 2000 stratified bootstrap replicates, *Threshold B* Gray zone method: 10% diagnosis tolerance

AUCs of PPV_Philips_, SVV_PiCCO_, PPV_PiCCO_, and PPV_LiDCC_ did not differ significantly in the cross-comparison. However, significant differences were observed for AUCs of PPV_Philips_, SVV_PiCCO_, PPV_PiCCO_, and PPV_LiDCO_ as compared with AUC of PVI (Table 3) but not for SVV_FloTrac_ and SVV_LiDCO_.

**Table 3:** Comparison of AUCs

*P*-values	PPV_Philips_	SVV_PiCCO_	PPV_PiCCO_	SVV_FloTrac_	SVV_LiDCO_	PPV_LiDCO_
SVV_PiCCO_	0.447					
PPV_PiCCO_	0.311	0.359				
SVV_FloTrac_	0.274	0.320	0.456			
SVV_LiDCO_	0.078	0.098	0.171	0.199		
PPV_LiDCO_	0.352	0.403	0.454	0.411	0.144	
PVI	0.015	0.020	0.043	0.053	0.219	0.034

*AUC* Area under the curve, *PPV* Pulse pressure variation, *PVI* Pleth variability index, *SVV* Stroke volume variation

## Discussion

Fluid responsiveness was tested by seven different functional hemodynamic parameters in mechanically ventilated patients after cardiac surgery. A wide range of areas under the ROC-curve (AUC’s) to predict fluid responsiveness was observed. SVV and PPV technologies differed in individual results but showed a good performance overall. The AUC’s to predict fluid responsiveness for SVV technologies were 0.78–0.91 and for PPV technologies 0.88–0.92. However, the non-invasive, functional parameter determined by the pulse oximeter, PVI showed only an AUC of 0.69.

Dynamic preload parameters such as SVV, PPV and PVI have been shown to be superior to traditional static indices in predicting a rise in stroke volume following a fluid challenge [14, 15]. Therefore dynamic indices are increasingly being used at the bedside to guide fluid therapy. These parameters are displayed by most of the currently available hemodynamic monitoring devices and their routine use is recommended by several treatment guidelines [16]. A prerequisite for their reliable performance is that patients are mechanically ventilated in a fully controlled mode with a tidal volume ≥ 8 mL kg^− 1^. Moreover decreased right ventricular function has to be excluded. Most of the guidelines recommend measuring one of these functional hemodynamic parameters but don’t differentiate between different technologies. Here we could show that each single technology has its own precision determing fluid responsiveness (Table 2, Fig. 1) and that the different technologies cannot be used interchangeably (Table 3).

However, the immediate postoperative phase after cardiac surgery on the ICU is characterized by rapid changes in cardiovascular dynamics. Only little data are available on functional hemodynamic variables to guide fluid therapy in this period and so far, evidence is scarce to show that volume management guided by functional hemodynamic variables should be more beneficial than standard of care [2]. Functional hemodynamic parameters have been evaluated in various clinical settings and results regarding accuracy, trending capabilities and fluid responsiveness have varied considerably [17]. The performance of these variables was reported to be reliable in controlled and stable conditions, e.g. after induction of anesthesia or during controlled ventilation in the ICU. Conversly, more rigorous protocols exposing patient to rapid changes in hemodynamics have shown major limitations in performance [18–20]. This might explain why the present study shows that absolute values of dynamic variables including gray zones were higher in contrast to previous studies [11, 21].

In our study, we used calibrated (PiCCO_2_) and un-calibrated (LiDCO_Rapid_, FloTrac) pulse-contour analysis to provide values for SVV and PPV. In addition, we measured PPV derived from routine intraarterial blood pressure monitoring (Philips) and PVI that was derived from pulse oximeter (Masimo). SVV and PPV of all systems are calculated from the minimal and the maximal SV (SV_min_, SV_max_) and the minimal and maximal PP (PP_min_, PP_max_) during the ventilator cycle using the following equations respectively: *SVV (%) = (SV*_*max*_ *- SV*_*min*_*) / SV*_*mean*_ and *PPV (%) = (PP*_*max*_ *- PP*_*min*_*) / PP*_*mean*_. While all systems derive PP directly from the arterial pressure waveform, they differ in SV determination and the time window that is required for the assesment of these functional hemodynamic parameters [6–8].

Using the PiCCO method, SV is calculated from the area under the systolic part of the arterial pressure waveform. In order to continuously display SV, the calibration by transpulmonary thermodilution is required that also allows for an adjustment of aortic compliance. For SVV calculation, the average of four extreme SV values during 30 s is assessed [22]. The LiDCO systems uses a so-called pulse power analysis based on the principle of mass and power conservation with a pressure dependent correction of compliance to measure SV. SVV is beeing assessed during a 20 s interval [23]. SV determination by the FloTrac system uses an algorithm assuming that the influence of pulse pressure to SV is proportional to the standard deviation of pulse pressure. It considers the influences of vascular resistance and compliance. Again, a time window of 20 s is required to assess SVV [19]. The knowledge of these different technologies helps to understand why one specific device measuring functional hemodynamic parameters performs differently compared to another in certain clinical situation. As we show in the present study, different technologies measuring the same functional hemodynamic variable show different performance.

PVI is a measure of the dynamic changes in the pulse oximeter signal (i.e. the pulse index) that occur during the respiratory cycle. The pulse index reflects the amplitude of the pulse oximeter waveform and is calculated as the pulsatile infrared signal (AC or variable component), indexed against the non-pulsatile infrared signal (DC or constant component). The infrared signal is used because it is less affected by changes in arterial saturation than the red signal. A recent meta-anaylsis described PVI equally effective for predicting fluid responsiveness compared to SVV and PPV in ventilated adult patients. In contrast to our results, the pooled performance was much better: the AUC for identification of fluid responders was 0.85 [24]. One possible explanation for the observed difference in PVI performance might be the patient population studied. We studied fluid responsiveness in postoperative patients after cardiac surgery and most of our patients were on norepinephrine. As shown previously, PVI is a weak predictor of fluid responsiveness in patients receiving norepinephrine [25].

The study has some limitations. This study was done in a small and selected group of cardiac surgery patients that were mechanically ventilated which precludes generalizability of our results. In addition, we used the ROC curve analysis to discriminate for fluid responsiveness. Although the ROC approach has been used in many studies dealing with fluid responsiveness, there is an unavoidable limitation since it artificially dichotomizes a continuous variable into a binary statistical index. Thereby, patients can only be categorized in fluid responders and non-responders according to their increase in SV after fluid loading. In the present study, our aim was to compare different technologies on how they perform predicting fluid response. This question can be answered by ROC curve anaylsis. However, in clinical reality, such a straight cutoff value (SV increase > 15%) is not always meaningful. Therefore, we additionally calculated the inconclusive zone (gray zone) where the accuracy of dynamic preload indices is limited in predicting positive fluid responsiveness [11, 26].

## Conclusions

Functional hemodynamic parameters of different invasive technologies, such as PPV and SVV performed well in predicting fluid responsiveness in mechanically ventilated patients after cardiac surgery except the non-invasive PVI technology. However, decision of fluid administration should not be based solely on the presence of preload responsiveness, but also on the presence of hemodynamic instability, peripheral hypoperfusion, clinical findings and the absence of high risk for fluid overload. All of the studied dynamic indices showed small but relevant differences between technologies.

## Abbreviations

AUC-ROC: Area und the receiver operating characteristic curve
EuroSCORE: European system for cardiac operative risk evaluation
ICU: Intensive care unie
PP: Pulse pressure
PPV: Pulse pressure variation
PVI: Pleth variability index
SV: Stroke volume
SVV: Stroke volume variation
